# Determining M2 macrophages content for the anti-tumor effects of metal-organic framework-encapsulated pazopanib nanoparticles in breast cancer

**DOI:** 10.1186/s12951-024-02694-z

**Published:** 2024-07-20

**Authors:** Zhijie Xu, Zhiyang Zhou, Xiaoxin Yang, Abhimanyu Thakur, Ning Han, Hai-Tao Li, Liu-Gen Li, Jun Hu, Tong-fei Li, Yuanliang Yan

**Affiliations:** 1 Department of Pathology, Xiangya Hospital, Central South University, Changsha, 410008 Hunan China; 2 Shiyan Key Laboratory of Natural Medicine Nanoformulation Research, Hubei Key Laboratory of Embryonic Stem Cell Research, School of Basic Medical Sciences, Hubei University of Medicine, Shiyan, 442000 Hubei China; 3 Department of Breast Surgery, Xiangya Hospital, Central South University, Changsha, 410008 Hunan China; 4 Clinical Research Center for Breast Cancer in Hunan Province, Changsha, 410008 Hunan China; 5 Department of Radiology, The Second Xiangya Hospital, Central South University, Changsha, 410008 Hunan China; 6 Department of Neurosurgery, Massachusetts General Hospital, Harvard Medical School, Boston, MA USA; 7 Department of Pharmacy, Xiangya Hospital, Central South University, Changsha, 410008 Hunan China; 8 National Clinical Research Center for Geriatric Disorders, Xiangya Hospital, Central South University, Changsha, 410008 Hunan China

**Keywords:** Pazopanib, Metal-organic framework, Immune microenvironment, M2-like macrophages, Breast cancer

## Abstract

Pazopanib (PAZ), an oral multi-tyrosine kinase inhibitor, demonstrates promising cytostatic activities against various human cancers. However, its clinical utility is limited by substantial side effects and therapeutic resistance. We developed a nanoplatform capable of delivering PAZ for enhanced anti-breast cancer therapy. Nanometer-sized PAZ@Fe-MOF, compared to free PAZ, demonstrated increased anti-tumor therapeutic activities in both syngeneic murine 4T1 and xenograft human MDA-MB-231 breast cancer models. High-throughput single-cell RNA sequencing (scRNAseq) revealed that PAZ@Fe-MOF significantly reduced pro-tumorigenic M2-like macrophage populations at tumor sites and suppressed M2-type signaling pathways, such as ATF6-TGFBR1-SMAD3, as well as chemokines including CCL17, CCL22, and CCL24. PAZ@Fe-MOF reprogramed the inhibitory immune microenvironment and curbed tumorigenicity by blocking the polarization of M2 phenotype macrophages. This platform offers a promising and new strategy for improving the cytotoxicity of PAZ against breast cancers. It provides a method to evaluate the immunological response of tumor cells to PAZ-mediated treatment.

**Supplementary Information:**

The online version contains supplementary material available at 10.1186/s12951-024-02694-z.

## Introduction

Breast cancer, one of the most malignant gynecologic cancers, remains the leading cause of cancer-related mortality among women worldwide [1]. Various therapeutic strategies, including surgical intervention, chemotherapy, and immunotherapy, have been used to mitigate the severity of breast cancer and potentially improve patient quality of life [2]. Despite significant therapeutic advances in recent years, many breast cancer patients still face largely incurable conditions and abysmal survival rates [3]. There is a critical need to develop novel therapeutic methods that can significantly improve the efficacy of treatment for this disease.

Molecular targeted therapy offers promising an alternative for clinical cancer management. Pazopanib (PAZ), a commercially available tyrosine kinase inhibitor, exhibits promising cytostatic and tumor-killing activities in several cancers, including breast cancer. Compared to other drugs, PAZ simultaneously inhibits multiple tyrosine kinases, including vascular endothelial growth factor receptor (VEGFR), platelet-derived growth factor receptor (PDGFR), and mast/stem cell growth factor receptor (c-Kit) [4, 5]. Administration of PAZ has been shown to significantly alter the viability of breast cancer cells and brain metastases both in vitro and in vivo [6]. Cheng et al. [7] reported an encouraging clinical response to PAZ in a breast cancer patient. However, its substantial side effects, such as diarrhea, nausea, cardiac dysfunction, and an increased risk of bleeding, limit its therapeutic application as an alternative treatment for breast cancer [8, 9].

Recent research developments in nanomedicine have suggested that nanoparticle-based technologies could revolutionize anti-breast cancer treatment by providing additional benefits and overcoming limitations. Materials integrated with carbon dots facilitate the controlled and targeted release of docetaxel within the tumor microenvironment, improving the concentration of docetaxel at the tumor site, minimizing side effects, and improving therapeutic sensitivity against breast cancer [10]. Furthermore, innovative nano-prodrugs such as hyaluronic acid-coated glutathione-responsive nanoparticles [11] have demonstrated superior fluorescence, enabling real-time monitoring of drug delivery into breast cancer tissues. Li et al. used engineered cancer cell membranes to encapsulate a ruthenium polypyridyl complex (RuPOP), discovering that this nano-platform significantly enhances the biological safety and anti-breast cancer efficacy of RuPOP [12]. Metal-organic framework (MOF)-based nanoparticles have been shown to promote targeted delivery of doxorubicin to breast cancer tissues and facilitate immunogenic cell death with minimal side effects [13]. Given the robust drug-loading capacity of nanoparticulate systems [14], there is a compelling interest in developing multifunctional nano-carriers to deliver PAZ to breast cancer tissues efficiently.

Metal-organic frameworks (MOFs) are porous materials of metal ions or clusters coordinated with organic ligands. Compared to other nanomaterials, MOF nanoparticles offer several advantages, including customizable pore sizes, functionalized pore surfaces, low density, and high surface areas. These properties make MOFs particularly attractive for various biomedical applications, particularly anti-cancer drug delivery [15, 16]. However, not all MOFs are suitable for drug delivery. Stability, biocompatibility, and drug-loading capacity require a thorough evaluation when selecting MOFs for such systems [17]. For example, by effectively integrating these properties, Fe (III)-based MOFs have emerged as an up-and-coming platform for delivering therapeutic agents to target tissues while minimizing off-target effects [18]. MIL-53(Fe) [19] and MIL-101(Fe) [20], two types of Fe (III)-based MOFs, have shown excellent chemical stability, high drug-loading capacity, and good safety profiles. Additionally, unlike in normal cells, the acidic environment (pH = 5.2) prevalent in cancer cells facilitates the release of ions from metal-containing MOFs [21]. These released ions can catalyze the production of lethal hydroxyl radicals from hydrogen peroxide within the tumor microenvironment, leading to oxidative damage in cancer cells [22]. Such features indicate that metal-containing MOFs are selectively cytotoxic to cancer cells and less harmful to normal cells. These findings suggest the significant potential of Fe (III)-based MOFs and the relevance of utilizing this nanotechnology to thoughtfully design anti-cancer strategies.

An ideal Fe (III)-based MOF nanostructure, PCN-224/Fe [23], was constructed using a Fe (III)-Tetrakis (4-carboxyphenyl) porphyrin (TCPP) complex, ZrOCl_2_·8H_2_O, and benzoic acid. PAZ was encapsulated in PCN-224/Fe through π-π interactions, resulting in the drug-loaded Fe-MOF nanoplatform, PAZ@Fe-MOF. To explore the impact of PAZ@Fe-MOF on the immune microenvironment in breast cancer, we performed high-throughput single-cell RNA sequencing (scRNAseq) to identify landscape changes. Our findings demonstrated a reduction in M2 macrophage subtypes specific for the anti-cancer functions of PAZ@Fe-MOF, an observation not previously described. These findings could offer new information on the biological functions of PAZ@Fe-MOF regarding the immune response in cancers.

## Materials and methods

### Preparation of PAZ@Fe-MOF

The nanosized Fe-MOF was synthesized according to a procedure reported by Zhang et al. [24]. Specifically, a solution of FeCl_3_-6H_2_O (4 mL, 15 mg/mL in dimethylformamide [DMF]) was mixed with a solution of Fe (III)-Tetrakis (4-carboxyphenyl) porphyrin (TCPP) (8 mL, 2.5 mg/mL in DMF) and benzoic acid (8 mL, 70 mg/mL in DMF). Subsequently, a PAZ solution (5 mL, 15 mg/mL in DMF) was gradually added to the mixture. This reaction mixture was heated under reflux at 90 °C for 5 h, followed by centrifugation and purification with DMF and ultrapure water. The prepared Fe-MOF was immersed in ethanol for 24 h to remove the retained DMF within the pores, thus mitigating the potential cytotoxic effects of DMF. The resulting product was vacuum-dried at room temperature to obtain the solid powder form of PAZ@Fe-MOF. Both Fe-MOF and the fluorescence-labeled variant PAZ@Fe-MOF-RhB were produced using the same synthesis processes to assess their intracellular and in vivo distribution.

### The characterization of PAZ@Fe-MOF

The size, metal junctions of iron ions and morphology of PAZ@Fe-MOF were observed by transmission electron microscopy (TEM). The chemical element contained in PAZ@Fe-MOF was analyzed by TEM elemental mapping and X-ray photoelectron spectroscopy (XPS). The hydrated size and zeta potential of PAZ@Fe-MOF were analyzed with a Malvern laser particle size analyzer. The Fourier transform infrared (FTIR) spectrum was applied to determine the characteristic vibrations of O = S = O on PAZ to prove that the PAZ was successfully loaded onto the Fe-MOF carrier. The crystal structure was characterized by X-ray diffraction (XRD), ultraviolet-visible (UV-vis) absorption spectrum, and thermogravimetric analysis (TGA). The specific surface area and average pore size of Fe-MOF and PAZ@Fe-MOF were measured using the N_2_ absorption and desorption assay. Red blood cells extracted from the goat’s blood were incubated with PAZ@Fe-MOF (2 h, 37 ℃) in the 24-well plates to analyze the hemolytic compatibility. PBS was added to the wells for negative control, and ultrapure water was added to the wells for positive control. The absorbance value (x) was calculated by measuring the absorbance at 540 nm of the erythrocyte supernatant after centrifugation. Red blood cell hemolysis rate = (x-negative/positive-negative)*100%.

### Orthotopic breast tumor models

The murine breast carcinoma 4T1 cells were cultured in Dulbecco’s modified Eagle’s medium (Biological Industries, Israel) containing 10% fetal bovine serum and incubated under the atmosphere with 5% CO_2_ at 37℃. Five-week-old female BALB/c mice were purchased from Hunan Sleck Jingda Laboratory Animal Company Limited. Appropriately 2 × 10^6^ 4T1 cells were injected into the right mammary fat pad to establish an orthotopic breast tumor model. In another orthotopic tumor model, primary tumors were generated by orthotopic implantation of 2 × 10^6^ MDA-MB-231 cells in the mammary fat pad of CB-17 SCID female mice. When tumors in these models reached the determined size (approximate 50 mm^3^), mice were randomly assigned to different treatment groups: (I) control groups, (II) Fe-MOF groups, (III) PAZ groups, and (IV) PAZ@Fe-MOF groups. Tumor volume was measured every 2 days and calculated using the following equation: volume = (length × width × width)/2. After 27 days of careful monitoring, the mice were euthanized, and the tumors were surgically dissected. Finally, tumor samples were photographed, weighed, and used for the following experimental analysis. The animal experiments were approved by the Ethics Committee of Central South University (No. CSU-2024-0085).

### In vivo distribution of PAZ@Fe-MOF

To analyze drug distribution and tumor-targeted properties, tumor-bearing mice were treated with PAZ@Fe-MOF-RhB (single administration by tail vein injection) for in vivo imaging assay (4, 8, 16, 24, and 48 h). The fluorescence intensity was detected in tumor tissues and vital organs. Alternatively, fixed tumor tissues were subjected to ultrasound sections, with nuclei subsequently stained using DAPI. The drug distribution (RhB fluorescence) in tumor tissues was observed using laser confocal microscopy.

### PET-CT imaging

Mice in each group were scanned with a small animal positron emission tomography-computed tomography (PET-CT) (Mediso, Hungary). Mice were administered an intravenous infusion of the ^18^F-FDG contrast agent and, 30 min later, were anesthetized with isoflurane (3% for induction and 1% for maintenance, using 100% O_2_ as a carrier) in an induction chamber equipped with a heating pad to maintain body temperature. After being secured to the examination board, the anesthetized mice underwent PET-CT scanning to acquire the images. Tumor size was measured three-dimensionally for each scan by delineating the maximum circular regions of interest (ROIs).

### scRNAseq

Peripheral blood and tumor specimens were harvested from the mice. For blood samples, after filtration through a 40 μm nylon cell strainer, red blood cells were lysed using 1× Red Blood Cell Lysis Buffer (Thermo Fisher Scientific, 00-4333-57). The remaining cell pellets were resuspended in pre-chilled 1× DPBS buffer (Solarbio, D1040) containing 2% fetal bovine serum (FBS, Thermo Fisher Scientific, SV30087.02). Tumor tissues were digested with 0.25% Trypsin (Hyclone, SH30042.01) and 10 µg/mL Recombinant DNase I (RNase-free) (TaKaRa, 2270B). Dissociated cells were labeled with CD45 MicroBeads (Miltenyi Biotec, 130-052-301) and sorted using MiniMACS™ Separators (Miltenyi Biotec, 130-042-102) and MS Columns (Miltenyi Biotec, 130-042-201). After sorting, at least 8 × 10^3^ CD45-positive cells were collected and resuspended in pre-chilled resuspension buffer. Cells were stained with AO/PI, and viability was assessed using a Countstar Fluorescence Cell Analyzer (Aber Instruments). Only single-cell suspensions with more than 80% viability were used for sequencing.

The sample processing was conducted using a 10×Genomics Chromium workstation and performed by Majorbio (Shanghai, China). Gel beads-in-emulsion (GEM)-encapsulated cells were lysed to release multiple barcode-targeted mRNAs for cDNA amplification and final library construction. The concentration and quality of sequencing-ready libraries were assessed using a High Sensitivity DNA Chip (Agilent) on a Bioanalyzer 2100 and the Qubit High Sensitivity DNA Assay (Thermo Fisher Scientific). The prepared libraries were sequenced on NovaSeq6000 (Illumina), targeting a minimum of 5 × 10^4^ reads per cell. The unique molecular identifier (UMI) raw data were processed using Cell Ranger 4.0, and low-quality cells with > 20% mitochondria-derived UMI counts were filtered out. The doublets identified by the DoubletFinder were also filtered. Subsequently, the count matrix was normalized with Seurat v.3.2.0. The data were first analyzed using a linear dimensionality reduction algorithm, principal component analysis (PCA). PCA was performed using the top 2,000 highly variable genes (HVGs) identified by FindVariableGenes from Seurat. Subsequently, the nonlinear dimensional reduction algorithm, t-distributed stochastic neighbor embedding (t-SNE), was utilized to visualize the sequencing data. Cell clusters were identified by SingleR, semi-supervised category identification and assignment (SCINA), and known marker genes. For each cluster, differentially expressed genes (DEGs) were identified using the Wilcoxon Rank-sum test by comparing them against the remaining clusters.

### Immunohistochemistry

Using pressure cooker heat, the exposed antigens of paraffin-embedded tumor tissues were repaired with sodium citrate buffer for 30 min. Sections were incubated with indicated primary antibodies at 37℃ for 1 h. Subsequently, these sections were incubated with secondary antibodies for 30 min. The immunohistochemistry signal was examined with a 3,3′-diaminobenzidine (DAB) solution and confirmed by two independent pathologists. The primary antibodies were Cd163 (1:200, 16646-1-AP, Proteintech, United States) and mannose receptor C-type 1 (Mrc1, 1:200, ab300621, Abcam, United States).

### M2-type chemokine analysis

ELISA was used to explore the effects of PAZ@Fe-MOF on the production of M2-type chemokines (C-C motif chemokine ligand 17 [CCL17], CCL22, and CCL24) and anti-inflammatory factors (interleukin 10 [IL10] and transforming growth factor beta [TGF β]) in tumor tissue homogenates. Tumor specimens were placed and homogenized in sterile PBS containing a protease inhibitor cocktail. After sonication and centrifugation, the supernatant of the tissue lysates was collected and stored frozen at -80℃. The supernatant was subjected to ELISA mouse cytokine assays per the manufacturer’s instructions. ELISA kits for M2-type chemokines were as follows: CCL17 (Cloud-Clone, SEB571Mu), CCL22 (Cloud-Clone, SEA091Mu), CCL24 (Cloud-Clone, SEA695Mu), TGF β (Cloud-Clone, SEA134Mu) and IL10 (Cloud-Clone, SEA056Mu).

### Western blot

The tumor samples were lysed and subjected to Western blot assays. Briefly, tissue lysates (50 µg) were separated by 10% SDS-PAGE and transferred to methanol-activated PVDF membranes. For the detection of M2-type signaling molecules, anti-activating transcription factor 6 (ATF6, Proteintech, 24169-1-AP), anti-transforming growth factor beta receptor 1 (TGFBR1, Proteintech, 30117-1-AP), anti-SMAD3 (Proteintech, 66516-1-Ig), anti-Phospho-SMAD3 (Zenbio, 380,775), anti-thioredoxin domain containing 5 (TXNDC5, Proteintech, 19834-1-AP), and anti-β-actin (Proteintech, 66009-1-Ig) were used.

### Quantitative RT-PCR

Tumor specimens were processed for RNA isolation using TRIzol reagent (Invitrogen, 15596-026), followed by cDNA synthesis with the SuperScript III First-Strand Synthesis System (Invitrogen, 18,080,051). Quantitative RT-PCR (qRT-PCR) was conducted using iTaq SYBR Green Supermix with ROX (Bio-Rad, 1,725,850) on a CFX96 Real-Time PCR Detection System. The primers for qRT-PCR were as follows: ATF6 (forward: 5′-TCTCCTCGGATGAGCAGGG-3′ and reverse: 5′-CTTCCCGAAGGGGTTCCAT-3′), TGFBR1 (forward: 5′-AGATCGCCCTTTCATTTCAG AG-3′ and reverse: 5′-GCAGTGGTAAACCTGATCCAGA-3′), SMAD3 (forward: 5′-TCTCCCCGAA TCCGATGTCC-3′ and reverse: 5′-GCTGGTTCAGCTCGTAGTAGG-3′), TXNDC5 (forward: 5′-TGATTCCGACGTGTGCTCTG-3′ and reverse: 5′-GGCTCCTCGTTCAGTGTCTG-3′) and β-actin (forward: 5′-CATGTACGTTGCTATCCAGGC-3′ and reverse: 5′-CTCCTTAATGTCACGCACGAT-3′).

### Flow cytometry

Tissues collected from orthotopic breast tumor models were harvested from the above-mentioned groups. The dissociated cells were marked with CD45 MicroBeads and isolated using a mouse CD45 MicroBeads kit (Miltenyi Biotec). Cell suspensions were centrifuged at 400 g for 5 min at 4 °C, and the supernatants were discarded. Cell pellets were incubated with APC anti-mouse Cd163 Monoclonal Antibody (eBioscience™, TNKUPJ) and FITC anti-mouse Mrc1 (Biolegend, 068C2) antibody on ice, in the dark, for 20 min. The cell pellets were re-suspended in 300 µL of flow buffer and filtered through a 40 μm mesh. The samples were detected using a flow cytometer, and data were analyzed using FlowJo V10 software (FlowJo, LLC).

### M2 macrophage infiltration assays

Macrophage M2 infiltration assays were conducted as previously described [25]. In the Transwell plate (8 mm, Corning), 2.5 × 10^5^ IL-4/IL-13-stimulated THP-1 cells were seeded with serum-free media in the upper chamber, while 2.5 × 10^5^ MDA-MB-231 breast cancer cells were seeded with the complete medium in the bottom plate. After a 24-h coincubation at 37 °C, THP-1 cells in the upper chamber were fixed in 4% formalin and stained with 0.3% crystal violet. The number of infiltrated M2-like macrophages was quantified in three randomly selected fields from each membrane.

### Detection of uptake and co-location of PAZ@Fe-MOF

IL-4-stimulated RAW264.7 cells (M2-like macrophages) seeded on the slide were treated with PAZ@Fe-MOF-RhB. After washing with PBS, cells were fixed before staining with ER-tracker (FITC fluorescence), and Hoechst 33,342. The intracellular uptake of PAZ@Fe-MOF and its colocalization with the endoplasmic reticulum (ER) were assayed by laser confocal microscopy. PAZ@Fe-MOF uptake was quantitatively detected in RAW 264.7 cells by flow cytometry.

### Statistical analysis

Statistical analysis of data from two groups was performed using the Student’s t-test in GraphPad Prism 8.3.9, and all data are presented as mean ± standard deviation (SD). For the single-cell RNA sequencing (scRNAseq) statistical analyses, the R package version 4.2.0 was utilized. A *p*-value of less than 0.05 was considered statistically significant.

## Results

### Characterization of PAZ@Fe-MOF

**Fig. 1:**
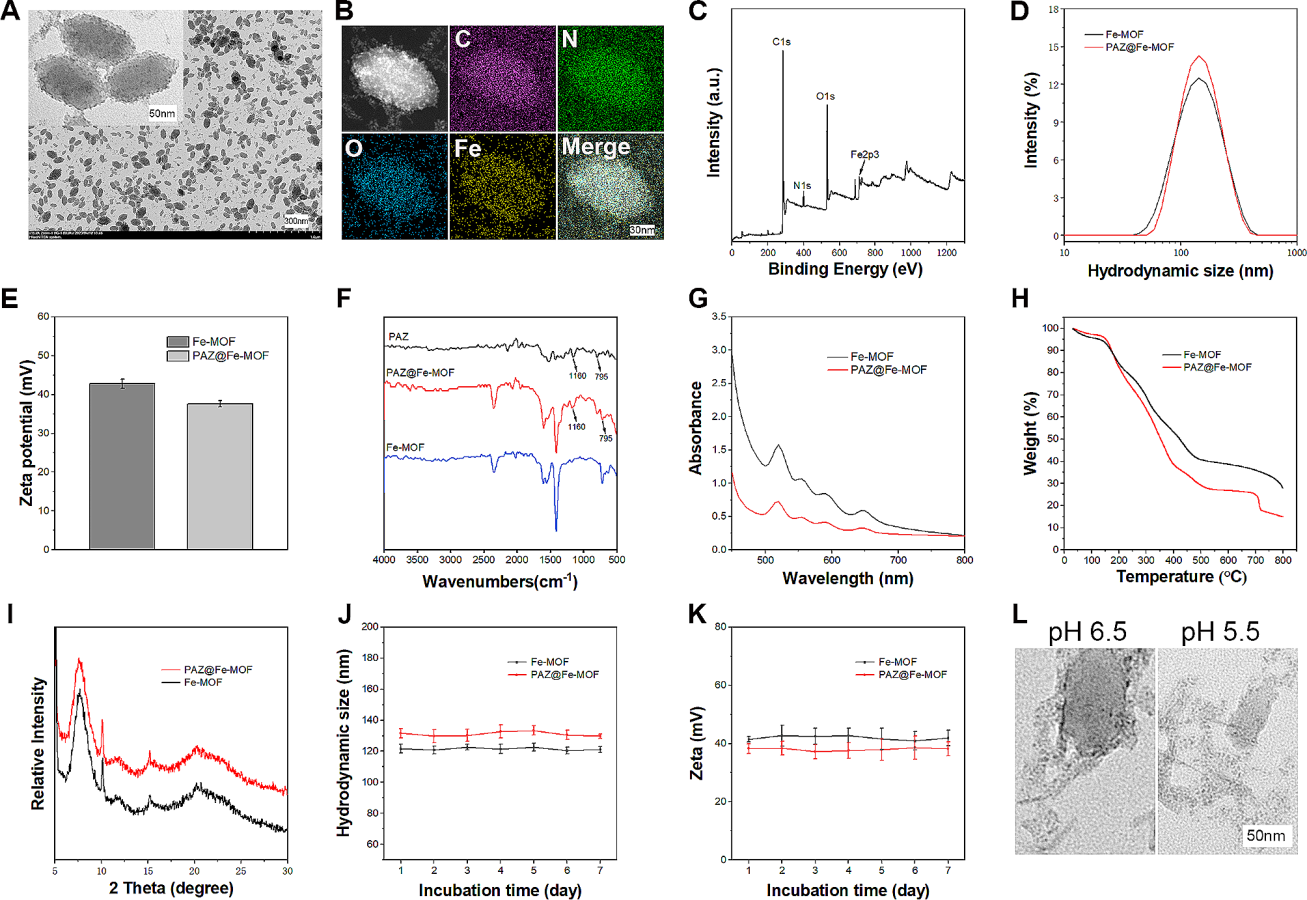
Characterizations of PAZ@Fe-MOF. (**A**) The TEM observation indicated that the successful preparation of PAZ@Fe-MOF. The particle size was about 100 nm. Pronounced metal junctions of iron ions on its surface could be observed. (**B**) The elemental mapping of PAZ@Fe-MOF. (**C**) The XPS detection results of PAZ@Fe-MOF for the elemental analysis. (**D**) The hydordynamic size of PAZ@Fe-MOF was about 122.6 nm, as detected by malvern laser particle size analyzer. (**E**) The zeta-potential of Fe-MOF and PAZ@Fe-MOF was measured by malvern laser particle size analyzer. (**F**) The FTIR spectrum analysis of PAZ@Fe-MOF. (**G**) UV-vis assay identified the crystal structure of Fe-MOF and PAZ@Fe-MOF. (**H**) The TGA analysis of Fe-MOF and PAZ@Fe-MOF. (**I**) X-Ray diffraction (XRD) experiments confirmed the Fe-MOF and PAZ@Fe-MOF crystal pattern. (**J**) The hydrated particle size of prepared nanoparticles varied little within 7 days. (**K**) The Zeta-potential of Fe-MOF and PAZ@Fe-MOF kept unchanged within 7 days. (**L**) The collapse of PAZ@Fe-MOF nanoparticles under different pH conditions detected by TEM

PAZ@Fe-MOF showed a homogeneous elliptical shape with pronounced iron ion metal junctions on its surface (Fig. 1A). The average size of the prepared nanoparticles was approximately 100 nm. The chemical elemental composition of PAZ@Fe-MOF was analyzed using visual elemental mapping and XPS analysis, revealing the presence of elements of C, N, O, and Fe (Fig. 1B-C). Hydrated particle sizes of Fe-MOF and PAZ@Fe-MOF were measured at 122.6 ± 2.8 nm and 130.3 ± 3.1 nm, respectively, before and after PAZ loading (Fig. 1D), as determined by a Malvern laser particle size analyzer. The zeta potentials of the nanoparticles were recorded at 42.8 ± 1.2 mV (Fe-MOF) and 37.6 ± 0.8 mV (PAZ@Fe-MOF), respectively (Fig. 1E). FTIR spectroscopy confirmed the successful characterization of PAZ-loaded Fe-MOF, showing significant vibrations at 795 and 1160 cm^− 1^, attributed to the characteristic vibrations of O = S = O in PAZ (Fig. 1F). UV-visible absorption spectra (Fig. 1G), TGA (Fig. 1H), and XRD (Fig. 1I) analyses indicated that the crystal structures of Fe-MOF remained unchanged before and after PAZ loading. Stability tests showed that Fe-MOF and PAZ@Fe-MOF maintained stable hydrated particle sizes and zeta potentials for 7 days (Fig. 1J-K). The loading and encapsulation efficiency of PAZ in PAZ@Fe-MOF were determined to be 23.80 ± 2.43% and 20.38 ± 3.09%, respectively (Table S1). N_2_ adsorption-desorption assays characterized the porous properties of the nanoparticles. The BET models showed a significant decrease in surface area after PAZ loading, which confirmed PAZ incorporation into the porous networks (Figure S1A). The hierarchical pore structure was further evident from the Barret-Joyner-Halenda (BJH) pore size distribution (PSD) curve, with a clear peak indicating mesopores in both Fe-MOF and PAZ@Fe-MOF, which almost disappeared after PAZ encapsulation (Figure S1B). PAZ@Fe-MOF exhibited partial collapse at a pH of 6.5 and further collapse at a pH of 5.5, indicating drug release in the tumor microenvironment (Fig. 1L). Additionally, the release of PAZ under acidic tumor conditions was further demonstrated (Figure S2). These findings indicate that PAZ@Fe-MOF was successfully synthesized and can respond to the acidic microenvironment in cancer cells.

### The anti-growth effects of PAZ@Fe-MOF on breast cancer models

**Fig. 2:**
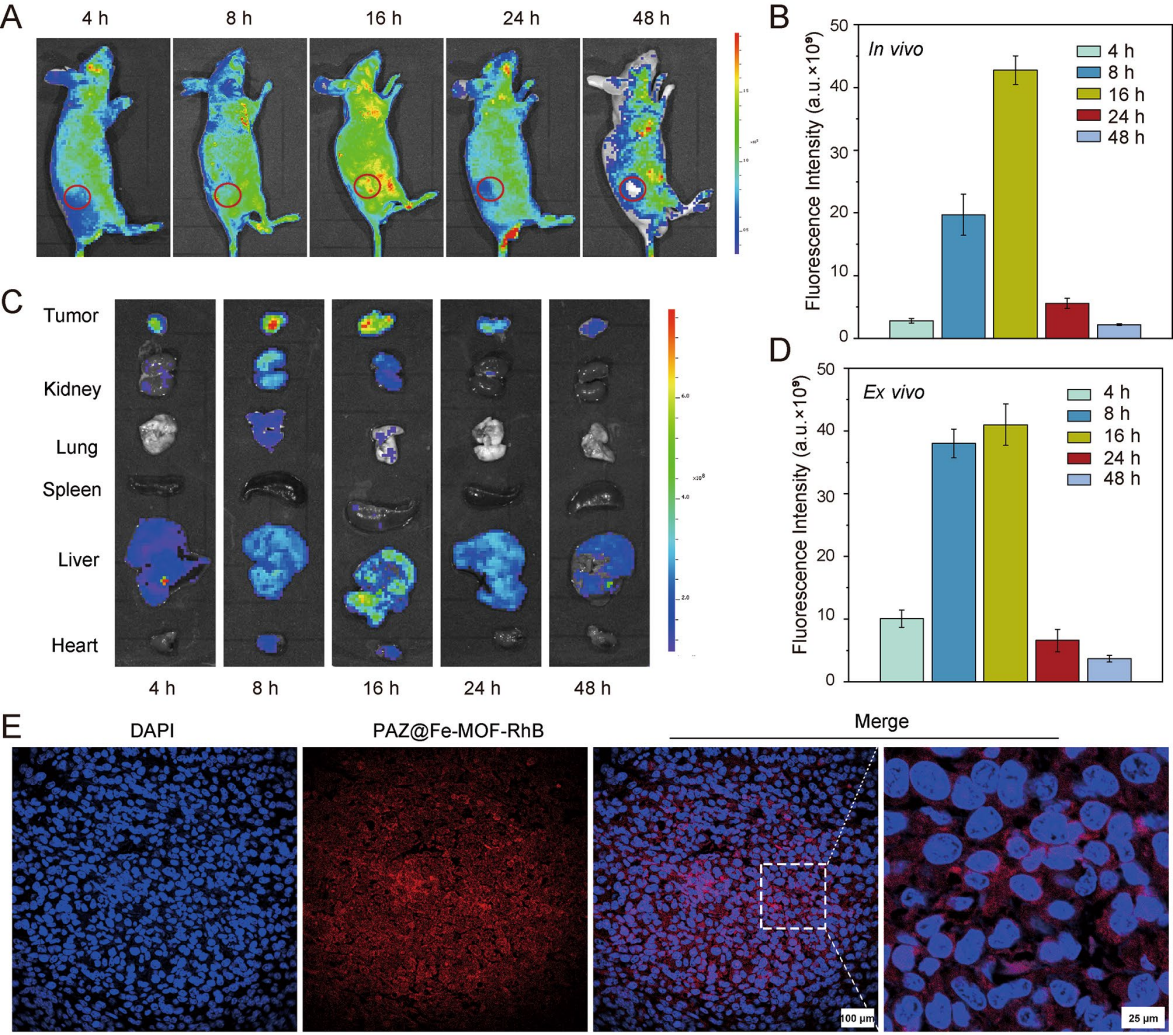
In vivo distribution of PAZ@Fe-MOF in orthotopic breast tumor models. (**A**) The distribution of PAZ@Fe-MOF-RhB was analyzed by in vivo imaging. The circle indicates tumor site. (**B**) Quantitative results of PAZ@Fe-MOF-RhB staining at tumor tissues from A. (**C**) The fluorescence distribution of PAZ@Fe-MOF-RhB in tumor tissue, kidney lung, spleen, liver and heart. (**D**) Quantitative results of PAZ@Fe-MOF-RhB staining at tumor tissues from C. (**E**) Representative images of PAZ@Fe-MOF-RhB staining in tumor tissue section

For porphyrin@MOFs, it is crucial to closely monitor the loading capacity, premature leakage, and self-quenching of porphyrins during the synthesis process of the nanomaterial [26]. Therefore, we synthesized the fluorescence-labeled PAZ@Fe-MOF (PAZ@Fe-MOF-RhB) to stably observe their tissue distribution in orthotopic breast tumor models using in vivo imaging techniques. As shown in Fig. 2A-D, PAZ@Fe-MOF-RhB demonstrated a pronounced distribution in tumor tissues 16 h after intravenous injection, with the highest accumulation observed in the liver. The fluorescence of PAZ@Fe-MOF-RhB significantly diminished 24 h post-injection. The presence of PAZ@Fe-MOF-RhB in the liver may facilitate detoxification and clearance processes [27]. These nanocarriers showed minimal distribution in other organs, such as the heart, lungs, spleen, and kidneys. Most importantly, drug fluorescence was directly observed in tumor tissues 16 h post-injection, as evidenced through fluorescence imaging (Fig. 2E), further confirming the targeted distribution of PAZ@Fe-MOF.

**Fig. 3:**
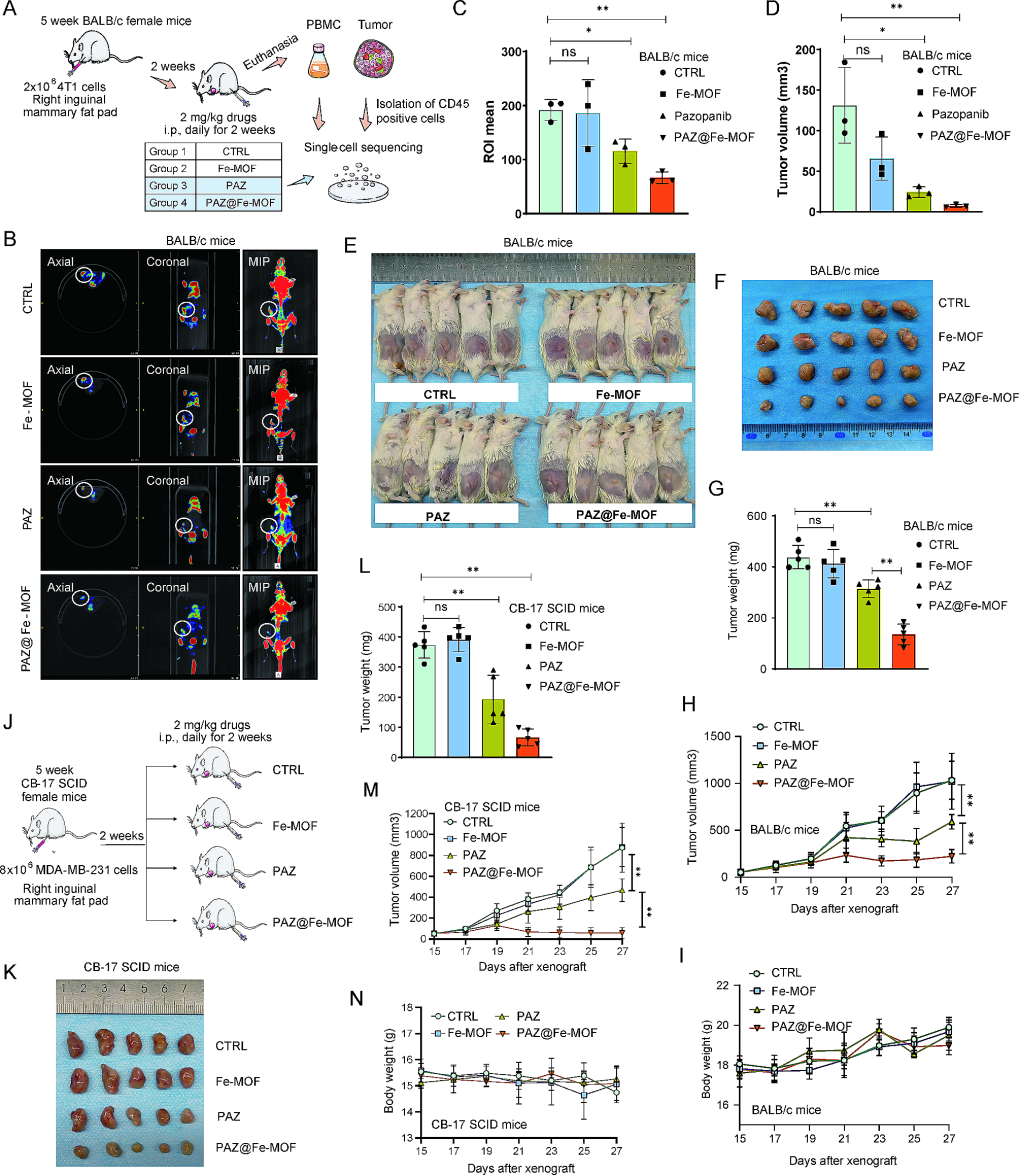
PAZ@Fe-MOF displayed the suppression of tumor growth in orthotopic breast tumor models. (**A**) Experimental design for the procedures of 4T1 orthotopic mice models. (**B**) The representative small-animal PET-CT images of tumoral ^18^F-FDG distribution. The circle indicates tumor site. (**C**) ROIs indicated the the tumor size. (**D**) The tumor volume measured by PET-CT. (**E-F**) The representative images of 4T1 orthotopic breast tumor models at the end of experiments. (**G**) The tumor weight in 4T1 orthotopic breast tumor models treated with Fe-MOF, PAZ or PAZ@Fe-MOF. (**H**) The tumor growth curves in 4T1 orthotopic breast tumor models treated with Fe-MOF, PAZ or PAZ@Fe-MOF. (**I**) The body weight curves in 4T1 orthotopic breast tumor models treated with Fe-MOF, PAZ or PAZ@Fe-MOF. (**J**) Experimental design for the procedures of MDA-MB-231 orthotopic tumor models. (**K**) The representative images of MDA-MB-231 orthotopic tumor models at the end of experiments. (**L**) The tumor weight in MDA-MB-231 orthotopic tumor models treated with Fe-MOF, PAZ or PAZ@Fe-MOF. (**M**) The tumor growth curves in MDA-MB-231 orthotopic tumor models treated with Fe-MOF, PAZ or PAZ@Fe-MOF. (**N**) The body weight curves in MDA-MB-231 orthotopic tumor models treated with Fe-MOF, PAZ or PAZ@Fe-MOF. ns: not significant, *: *p*-value < 0.05, **: *p*-value < 0.01

To assess the therapeutic potential of PAZ@Fe-MOF on breast cancer, we randomly assigned 4T1 orthotopic mouse models into four groups: (I) control, (II) free Fe-MOF-treated, (III) PAZ-treated, and (IV) PAZ@Fe-MOF-treated (Fig. 3A). Studies have indicated that PET-CT provides a non-invasive and quantitative method for tumor evaluation with enhanced sensitivity and specificity, offering advantages over other imaging modalities [28]. Moreover, in small-animal PET-CT images, the parameter of tumoral ^18^F-FDG distribution has been widely used to evaluate the tumor size after different treatments [29]. As shown in Fig. 3B-C, micro-PET-CT images showed increased ^18^F-FDG distribution in the control and free Fe-MOF-treated groups. In contrast, treatment with PAZ alone decreased ^18^F-FDG distribution, while PAZ@Fe-MOF treatment significantly reduced it. Additionally, PET-CT scans revealed a significant reduction in tumor volume in the PAZ@Fe-MOF-treated groups compared to those treated with free PAZ (Fig. 3D). Tumors from the 4T1 models were surgically excised and photographed (Fig. 3E-F). Compared to free PAZ, PAZ@Fe-MOF treatment markedly reduced tumor weight and volume, affirming its enhanced therapeutic efficacy (Fig. 3G-H). Importantly, minimal changes in body weight were observed in mice treated with PAZ@Fe-MOF (Fig. 3I). Hematoxylin-eosin (H-E) staining showed no pathological changes in vital organs such as the heart, liver, spleen, lung, and kidney (Figure S3). PAZ@Fe-MOF (PAZ concentration up to 500 µg/mL) exhibited minimal cytotoxicity to red blood cells (Figure S4), demonstrating favorable hemolytic compatibility and indicating that it does not compromise blood cell integrity during distribution. These findings suggest the negligible adverse effects of PAZ@Fe-MOF in vivo.

To further validate the cytotoxic effects of PAZ@Fe-MOF observed in the 4T1 model, we used a second mouse model using human breast cancer cells, MDA-MB-231, in CB-17 SCID female mice (Fig. 3J). As expected, PAZ@Fe-MOF nanoparticles demonstrated robust antitumor efficacy, as evidenced by significantly reduced tumor weight and volume (Fig. 3K-M). Treatment with PAZ@Fe-MOF resulted in minimal changes in body weight (Fig. 3N). These data show the clinical relevance and safety of PAZ@Fe-MOF as a nanodrug for treating breast cancers.

### Single-cell profiling in blood revealed the roles of monocytes in response to PAZ@Fe-MOF

Having established its therapeutic effects, we sought to further assess how individual cell populations in the blood were affected by PAZ@Fe-MOF. Emerging studies indicate that single-cell RNA sequencing (scRNAseq) has become a crucial technology for characterizing the complex signaling networks that control the biological activity of nanomaterials [30]. We isolated peripheral blood mononuclear cells (PBMCs) from mice treated with PAZ@Fe-MOF and free PAZ and then performed single-cell sequencing. Data visualization using t-SNE identified several different cell types, including granulocytes, monocytes, and T cells (Figure S5A). Compared to the PAZ-treated groups, PAZ@Fe-MOF treatment significantly reduced monocyte prevalence and increased granulocyte prevalence. The infiltration of T cells was abundant, although their concentration did not change significantly (Figure S5B). Monocytes emerged as a candidate cluster with signifcantly enriched gene expression (Figure S5C).

**Fig. 4:**
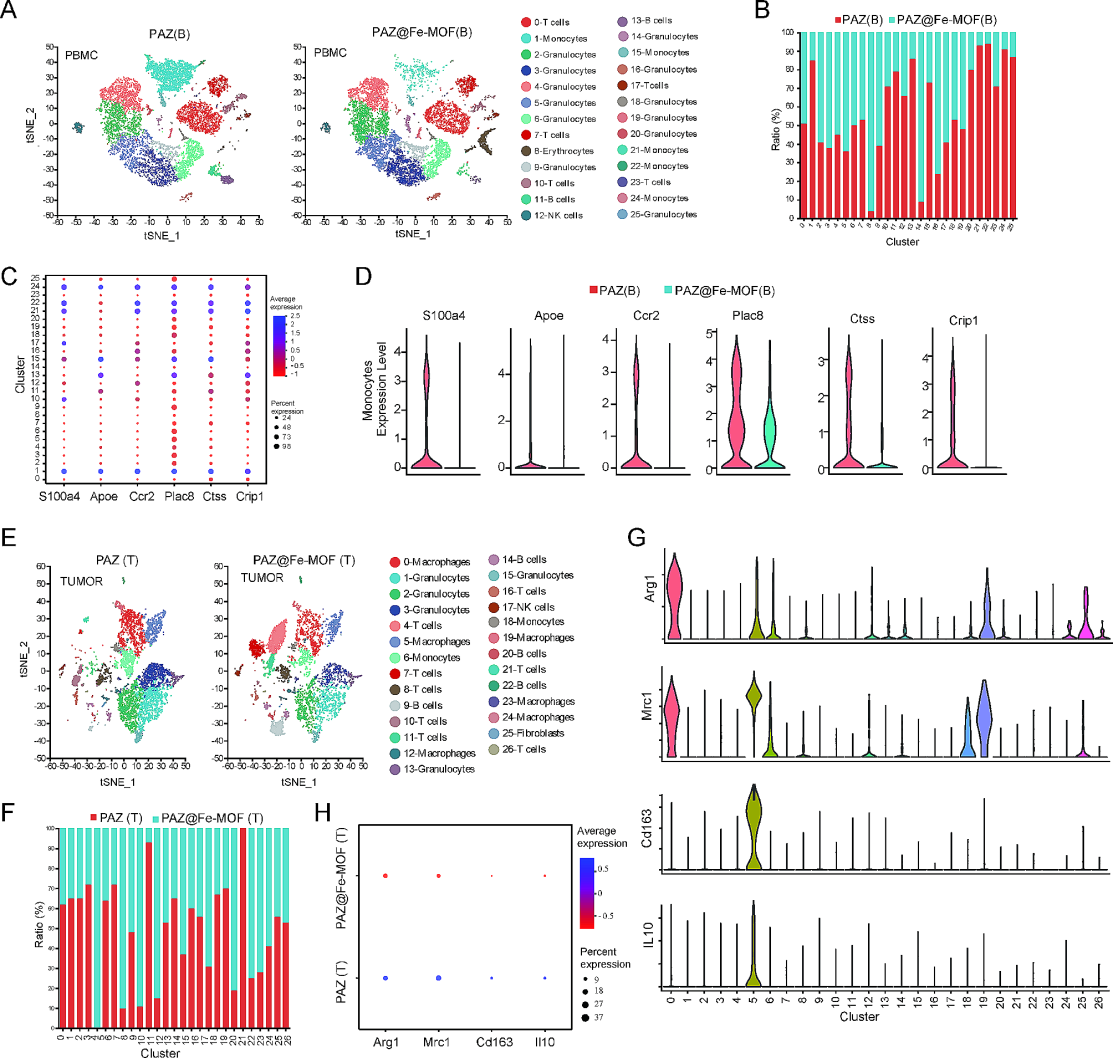
scRNAseq identified the effect of PAZ@Fe-MOF on monocyte-derived macrophages. (**A-B**) The changes of twenty-six subpopulations in blood after PAZ@Fe-MOF treatment. (**C**) Bubble plots indicated the expression of monocytic makers in each single cell cluster. (**D**) Violin plots indicated the changes of monocytic makers after PAZ@Fe-MOF treatment. (**E-F**) The changes of different cell clusters in tumor tissues after PAZ@Fe-MOF treatment. (**G**) Violin plots of M2-like macrophage makers for each cluster. (**H**) Bubble plots indicated the changes of M2-like macrophage makers after PAZ@Fe-MOF treatment

We conducted a more detailed cluster analysis based on specific DEGs, identifying 26 subpopulations (Figure S5D). Clustering analysis highlighted distinct transcriptional profiles among these subpopulations (Figures S5E-F). Subtypes 1, 10, 11, 12, 13, 15, 20, 21, 22, 23, 24, and 25 were most significantly down-regulated in the PAZ@Fe-MOF-treated groups compared to the free PAZ-treated groups, with subtype 1 (monocytes) being the most prevalent and abundant (Fig. 4A-B). This subtype exhibited high levels of transcripts associated with monocyte function, such as S100 calcium-binding protein A4 (S100a4), apolipoprotein E (Apoe), C-C motif chemokine receptor 2 (Ccr2), placenta-specific 8 (Plac8), cathepsin S (Ctss), and the cysteine-rich protein 1 (Crip1) (Fig. 4C). Hoffmann et al. [31] identified S100A4-positive inflammatory monocytes in convalescent COVID-19 patients. Tsuboi et al. [32] recognized Plac8 as a marker involved in the pathogenesis of adult-onset Still’s Disease. Other genes, such as Apoe [33], Ccr2 [34], Ctss [35], and Crip1 [36], have also been identified as signature markers of monocytes. In particular, the administration of PAZ@Fe-MOF down-regulated these six monocytic biomarkers (Fig. 4D, Figure S5G). We then extracted the subtype 1 specific marker genes (monocytes) to conduct a KEGG enrichment analysis, revealing that these monocytic biomarkers mainly participate in the regulation of several immunological pathways, including the positive regulation of the inflammatory response **(Figure **S5H). These peripheral blood scRNAseq data suggest the possible immunological functions of monocytes in response to PAZ@Fe-MOF treatment.

### Single-cell profiling in tissues revealed the effect of PAZ@Fe-MOF on M2 macrophages

Given that circulating monocytes serve as precursors to macrophages and dendritic cells (DCs) [37], we evaluated intratumoral abundances of macrophages, DCs, and other cells in mice treated with PAZ@Fe-MOF or free PAZ. After treatment, dissociated cells from tumor tissues were isolated using CD45 MicroBeads and analyzed with scRNAseq. RNA profiling successfully identified 27 cell clusters from all CD45 + cells, annotated into seven cell types: macrophages, granulocytes, T cells, monocytes, B cells, NK cells, and fibroblasts. DCs were not captured significantly in the scRNAseq samples (FiguresS6A-B). Clustering analysis highlighted different transcriptional profiles among these clusters (FiguresS6C-D). The prevalence of clusters 0, 1, 2, 3, 5, 6, and 10 decreased in tumors treated with PAZ@Fe-MOF (Fig. 4E-F). In contrast, clusters 4, 7, 9, 11, 20, 22, and 23 exhibited an increased prevalence after PAZ@Fe-MOF administration. Among these, cluster 5 was significantly abundant, characterized by high levels of M2 macrophage biomarkers such as arginase 1 (Arg1), Mrc1, Cd163, and IL10 [38, 39] (Fig. 4G, S6E). In response to PAZ@Fe-MOF treatment, there was a significant down-regulation of these four M2 markers (Fig. 4H). We also analyzed the M1 macrophage biomarkers in different clusters and found that cluster 23 showed high levels of M1 biomarkers, including Ccr7 and Il12b [40] (Figure S6F). After PAZ@Fe-MOF treatment, these M1 markers were obviously up-regulated (Figure S6G). However, cluster 23, identified by scRNAseq, was unsuitable for further analysis due to the small number of cells. The scRNAseq analysis suggests that M2-like macrophages play a crucial role in mediating the therapeutic effects of PAZ@Fe-MOF.

### The pseudotime trajectory of monocyte-macrophage during PAZ@Fe-MOF treatment

Breast cancer is typically characterized by the infiltration of circulating monocyte-derived macrophages into tumor tissues, which influences tumorigenesis and therapeutic responses [41, 42]. To explore monocyte-to-macrophage differentiation during PAZ@Fe-MOF treatment, a pseudotime trajectory analysis was conducted using both blood cells and tissue cells. By integrating sequencing data from blood and tissue specimens, we annotated all cells into 29 clusters based on DEGs (Figure S7A). In particular, the prevalence of cluster 0 decreased significantly in blood and tumor samples treated with PAZ@Fe-MOF (Figure S7B). The top 10 transcription factors in cluster 0 were distinctively expressed compared to other clusters. Importantly, cluster 0 up-regulated several genes associated with monocytes and macrophages, including Apoe, Pf4, Mrc1, C-C motif chemokine ligand 8 (Ccl8) [43], complement C1q chain (C1qa) [44], and legumain (Lgmn) [45] (Figure S7C).

We identified nine monocyte-macrophage subpopulations (Figure S7D). Among them, subcluster 0 exhibited up-regulated monocyte markers such as S100a4, Apoe, Ccr2, Plac8, Ctss, and Crip1 (Figure S7E). In contrast, subcluster 3 showed up-regulation of M2 macrophage markers, including Arg1, Mrc1, Cd163, and IL10, and down-regulation of the M1 macrophage marker IL1b [46] (Figure S7F). We assessed the dynamic changes in gene expression along the pseudotime trajectory to elucidate monocyte-macrophage differentiation. Blood cells were located primarily at the beginning of the trajectory, whereas tumor cells appeared at the end (Figure S7G), supporting the transition from monocytes to macrophages [37]. Administration of PAZ@Fe-MOF resulted in decreased accumulation of subcluster 3 at the trajectory branches (Figure S7H), suggesting inhibition of M2 macrophage differentiation. This indicates a potential mechanism by which PAZ@Fe-MOF modulates the immune environment in breast cancer.

### Inhibition of M2 macrophage polarization by PAZ@Fe-MOF

**Fig. 5:**
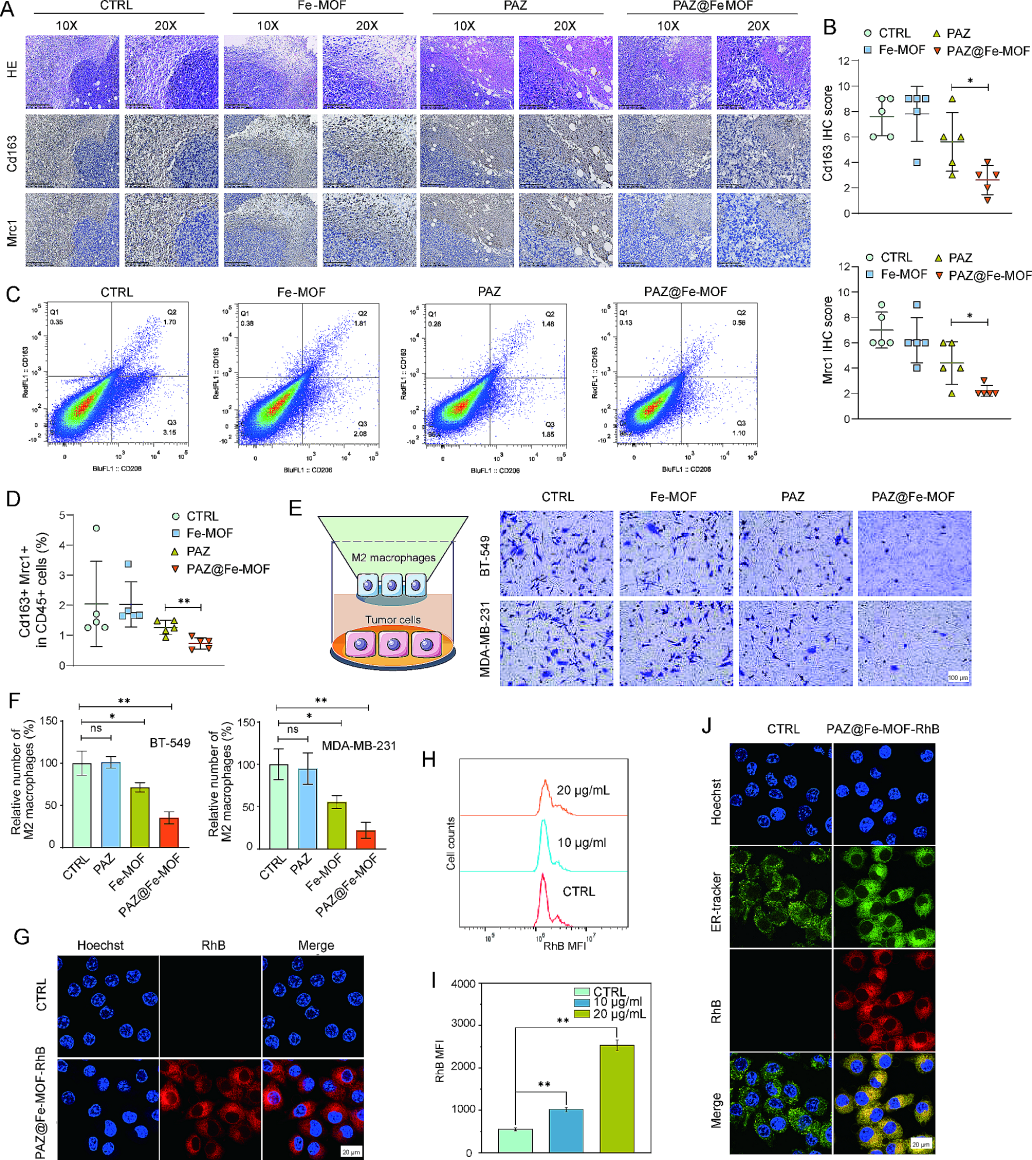
Validation of decreased M2-like macrophages following PAZ@Fe-MOF treatment. (**A**) Representative immunostaining images of Mrc1 and Cd163 on mice specimens. Scale bars are indicated. (**B**) Protein levels of Mrc1 and Cd163 were quantified in mice specimens with different treatment. (**C-D**) Flow cytometry indicated the effects of PAZ@Fe-MOF on the tumor-infiltrating Cd163 + Mrc1 + cells. (**E-F**) Infiltration of M2-like macrophages in human breast cancer cells MDA-MB-231 treated with the indicated conditions. (**G**) PAZ@Fe-MOF-RhB nanoagents could be absorbed by IL-4-stimulated RAW264.7 cells. (**H-I**) The mean fluorescence intensity was quantified. (**J**) ER was labeled by the ER-tracker. In IL-4-stimulated RAW264.7 cells, the co-localization of PAZ@Fe-MOF with ER were observed with confocal microscopy. *: *p*-value < 0.05, **: *p*-value < 0.01

To confirm the inhibitory effects of PAZ@Fe-MOF on M2 macrophage polarization, we performed immunohistochemistry staining for Mrc1 and Cd163 on formalin-fixed, paraffin-embedded mouse specimens. Representative immunostaining images of Mrc1 and Cd163 are displayed in Fig. 5A. Expression levels of Mrc1 and Cd163 in the PAZ@Fe-MOF-treated groups were significantly lower than those in the PAZ-treated groups (Fig. 5B). Additionally, flow cytometry analysis showed that PAZ treatment mildly impaired the infiltration of Cd163 + Mrc1 + M2-like macrophages, whereas PAZ@Fe-MOF treatment induced additive effects, further reducing their presence (Fig. 5C-D). M2 macrophage infiltration assays indicated that PAZ@Fe-MOF treatment significantly decreased the infiltration of IL-4/IL-13-stimulated THP-1 cells (M2-like macrophages) into breast cancer tissues (Fig. 5E-F). PAZ@Fe-MOF nanoagents were effectively absorbed by IL-4-stimulated RAW264.7 cells (M2-like macrophages) (Fig. 5G-I), leading to the co-localization of PAZ@Fe-MOF with the endoplasmic reticulum (ER) in these cells (Fig. 5J). These findings suggest that the impaired infiltration of M2 macrophages might be critical for the anti-cancer activities of PAZ@Fe-MOF against breast cancer.

**Fig. 6:**
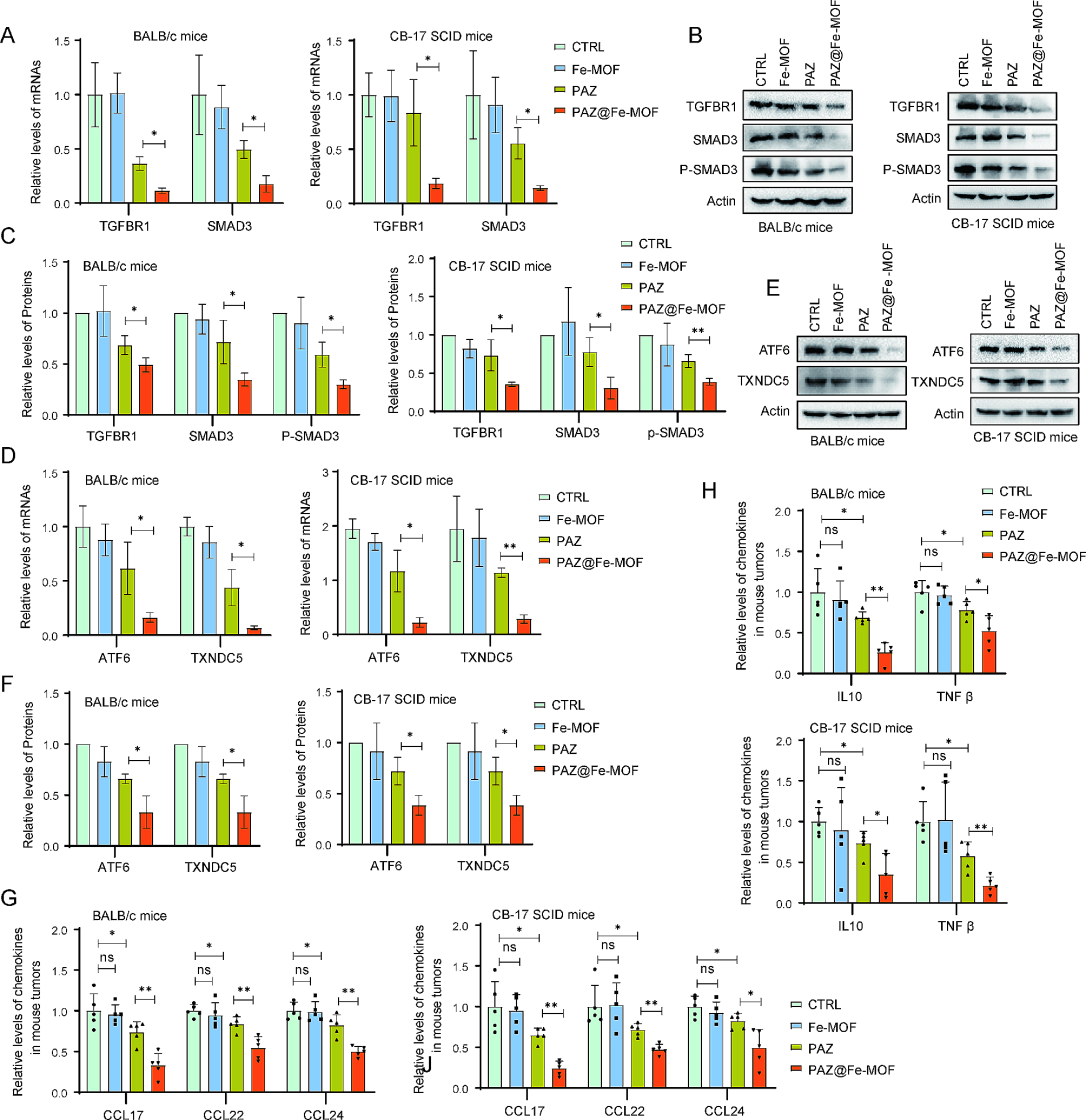
Inhibitory effect of PAZ@Fe-MOF on TGFBR1-SMAD3-ATF6-TXNDC5 signaling axis. (**A**) Syngeneic murine 4T1 and xenograft human MDA-MB-231 breast cancer models were treated with Fe-MOF, PAZ or PAZ@Fe-MOF. Tumor tissues were collected and processed for RNA isolation. The mRNA expression of TGFBR1 and SMAD3 was analyzed using qRT-PCR. (**B-C**) Tumor tissues were lysates and then blotted with indicated antibodies. (**D**) The mRNA expression of ATF6 and TXNDC5 in tumor tissues was analyzed using qRT-PCR. (**E-F**) Tumor tissues were lysates and then blotted with indicated antibodies. (**G-H**) The effect of PAZ@Fe-MOF on M2-type chemokines (CCL17, CCL22 and CCL24) and anti-inflammatory factors (IL10 and TGF β) in tumor tissue homogenates

To further explore the underlying molecular mechanisms of PAZ@Fe-MOF on M2 macrophage polarization, we extracted total RNA and proteins from tumor specimens in syngeneic murine 4T1 and human MDA-MB-231 xenograft breast cancer models. Increasing evidence indicates that the TGFBR1-SMAD3 signaling pathway plays a primary role in determining the polarization of M2 macrophages [47]. Upon treatment with PAZ@Fe-MOF, we observed a significant decrease in the expression of TGFBR1, SMAD3, and phosphorylated SMAD3 (Fig. 6A-C). This signaling pathway is known to upregulate the expression of the TXNDC5 ER protein through ATF6-dependent transcriptional control [48]. Given the co-localization of PAZ@Fe-MOF with the ER (Fig. 5J), we performed qRT-PCR and Western blot experiments, which revealed a significant downregulation of ATF6 and TXNDC5 after PAZ@Fe-MOF treatment (Fig. 6D-F). Furthermore, the secretion of anti-inflammatory mediators by M2 macrophages, such as CCL17, CCL22, CCL24, TGF-β, and IL-10 [49], was also significantly suppressed (Fig. 6G-H). These findings suggest that the inhibitory effect of PAZ@Fe-MOF on M2 macrophage polarization may be mediated by inhibiting the TGFBR1-SMAD3-ATF6-TXNDC5 signaling axis.

## Discussion

As a multitargeted tyrosine kinase inhibitor, PAZ exhibits dose-dependent inhibitory activity against several cancers, including breast cancer. In 2009, the U.S. Food and Drug Administration approved PAZ to manage patients with advanced renal cell carcinoma [50]. PAZ significantly enhances the production of intracellular reactive oxygen species and activates ER stress signaling, which subsequently triggers apoptosis in lung cancer cells, specifically NCI-H446 and NCI-H82 [51]. In renal cell carcinoma, IGFL2-AS1 knockdown has been shown to suppress the expression and activation of the androgen receptor, leading to the downregulation of vascular mimicry and increased efficacy of PAZ [52]. Additionally, PAZ administration has been found to dramatically stimulate the deactivation of PDGFR-positive astrocytes in the neuroinflammatory microenvironment, impairing brain metastases of cancer cells [53]. However, a phase II multicenter single-arm study reported several adverse events during PAZ treatment in patients with HER2-negative advanced breast cancer, including neutropenia, diarrhea, and hypertension [9]. Cheng et al. [7] also observed neoplasm recurrence and disease progression in some breast cancer patients after starting PAZ therapy, indicating resistance to PAZ. Therefore, it is urgently necessary to develop individualized strategies to maximize the sensitivity to PAZ treatment.

**Fig. 7:**
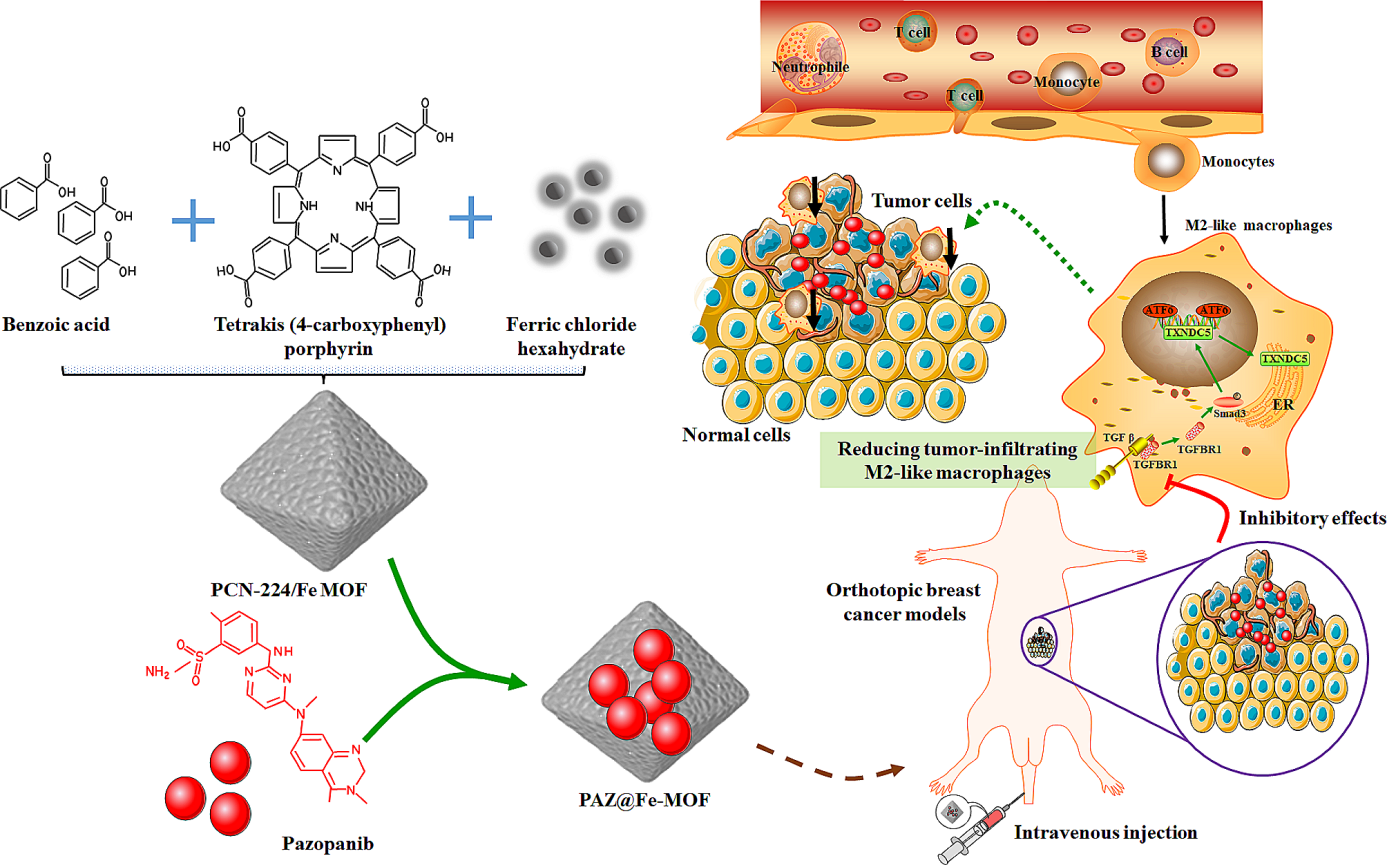
Schematic diagram of how PAZ@Fe-MOF nanoparticles affect the immunological microenvironment in breast cancers. Upon treatment with PAZ@Fe-MOF, the pro-tumor M2-like macrophage populations were significantly down-regulated in tumor sites, subsequently reversing the inhibitory immune microenvironment and blocking tumorigenicity

Nanotechnology-based therapies offer significant advantages that can help overcome the limitations of traditional anti-cancer drugs [54, 55]. Accordingly, various lipid nanocarriers have been used to enhance the antagonistic effects of PAZ against cancers. For example, PAZ-loaded nanoliposomes have been developed and shown to exhibit improved anti-cancer therapeutic efficacy [56, 57]. Recently, Nadaf et al. [58] constructed new PAZ-loaded solid lipid nanoparticles (Pazo-SLNs), demonstrating targeted absorption by lung cancer cells and enhanced sensitization of tumor cells to PAZ. Similarly, we developed self-assembled Fe (III)-based MOF nanoplatforms to carry PAZ (PAZ@Fe-MOF). In vivo experiments confirmed that PAZ@Fe-MOF treatment facilitated enhanced tumor-killing activities against breast cancers (Fig. 7). Importantly, Fe (III)-based MOFs offer advanced functionalities, such as a high PAZ-loading capacity and pH-responsive release, making them highly promising for PAZ delivery applications. However, their complex synthesis, higher costs, and potential biocompatibility concerns may limit their practicality compared to established and biodegradable lipid nanoparticles.

It should be noted that macrophage infiltration plays a crucial role in host immune responses, contributing to the maintenance of microenvironment homeostasis. Subsequent studies have shown the significant regulatory functions of tumor-infiltrating macrophages in both tumorigenesis and therapeutic efficacy [59, 60]. For example, in pancreatic ductal adenocarcinoma, the knockdown of CCCTC-binding factor (CTCF) resulted in the downregulation of specific biomarkers of M2-like tumor-associated macrophages (TAMs), such as Mrc1 and CD163, reducing invasion and metastasis capabilities [61]. In breast cancer cells (MCF7), melatonin treatment reversed the immunosuppressive microenvironment and inhibited tumorigenicity by increasing the M1/M2 macrophage ratio [62]. The direction of M1/M2 polarization in cancers can be influenced by various nanosized strategies to enhance therapeutic efficacy, including using iron-based nanoparticles [63]. For example, carboxymethylated β-glucan-loaded iron oxide nanoparticles (CMPTR/IONPs) effectively converted M2-like populations into M1 phenotypes, stimulating a cascade of anti-tumoral immune responses [64]. Ou et al. [65] used iron oxide nanoparticles to deliver recombinant adeno-associated viral type 2, forming ION-AAV2 nanoparticles. In vivo assays demonstrated that by reprogramming pro-tumor M2 phenotypes into anti-tumor M1 phenotypes, ION-AAV2 facilitated the release of proinflammatory cytokines and suppressed liver cancer growth. Similarly, Liu et al. [66] developed new ultrasmall superparamagnetic iron oxide nanoparticle-based molecular imaging nanoprobes, which quantitatively triggered the repolarization of M2 to M1 macrophages in tumors, activating the immune system and enhancing anti-cancer treatment efficacy. Consistent with these concepts, our analyses have shown that intravenous injection of PAZ@Fe-MOF effectively targeted and reduced M2 macrophage infiltration, exerting tumor-suppressing effects in breast tumor models.

## Conclusion

PAZ@Fe-MOF enhances anti-breast cancer immunity primarily by reducing intratumoral infiltration of M2 macrophages. The development of PAZ-encapsulated nanoplatforms, such as MOF-based nanoparticles, represents a promising approach to potentially eradicate breast cancers. This advance demonstrates the significance of targeted nanotherapy in improving cancer treatment outcomes.

### Electronic supplementary material

Below is the link to the electronic supplementary material.

Supplementary Material 1: Figure S1. The porous property of nanoparticles was characterized by N2 absorption and desorption assay. (A-B) The BET surface area (A) and BJH pore size distribution (B) were detected using N2 absorption and desorption assay.

Supplementary Material 2: Figure S2. The release of PAZ from PAZ@Fe-MOF nanoparticles under different pH conditions.

Supplementary Material 3: Figure S3. HE staining indicated the morphological changes of vital organs in orthotopic breast tumor models.

Supplementary Material 4: Figure S4. The hemolysis rate of the PAZ@Fe-MOF to the red blood cells was detected, which reflected the hemolytic compatibility of the prepared nanoparticles.

Supplementary Material 5: Figure S5. Single-cell profiling and cell typing in blood. (A) t-SNE visualization of cell types in peripheral blood from the mice treated with PAZ@Fe-MOF and free PAZ. (B) Pie chart indicated the changes of different cell clusters after PAZ@Fe-MOF treatment. (C) The numbers of characteristic genes in different cell clusters. (D) t-SNE indicated the twenty-six clusters from the peripheral blood. (E) Bubble plots indicated the top 3 specific genes in each cluster from the peripheral blood. (F) A heatmap indicated the top 10 specific genes in each cluster from the peripheral blood. (G) Bubble plots indicated the changes of monocytic makers after PAZ@Fe-MOF treatment. (H) The KEGG enrichment analysis of the DEGs between cluster 1 and other clusters.

Supplementary Material 6: Figure S6. scRNAseq identified the effect of PAZ@Fe-MOF on M2 macrophages in tissues. (A) t-SNE plots indicated seven cell types from all CD45+ cells. (B) t-SNE indicated the twenty-seven clusters from the tissues. (C) Bobble plot indicated the top 3 specific genes in each cluster from the tissues. (D) A heatmap indicated the top 10 specific genes in each cluster from the tissues. (E) The expression of M2-polarized macrophage makers in each cluster profiled on the t-SNE plots. (F) Bubble plots indicated the expression of M1-like macrophage makers in each single cell cluster. (G) Bubble plots indicated the changes of M1-like macrophage makers after PAZ@Fe-MOF treatment.

Supplementary Material 7: Figure S7. Reconstructing the pseudotime trajectory of monocyte-macrophage during PAZ@Fe-MOF treatment. (A) t-SNE plots of twenty-nine cell types from blood and tissue specimens. (B) Cell percentages of each cluster were shown in the histograms. (C) The top 10 transcription factors in clusters 0 compared with other clusters. (D) t-SNE plots of nine monocyte-macrophage subpopulations from clusters 0. (E) Bubble plots indicated the expression of monocytic makers in each single cell cluster. (F) Bubble plots indicated the expression of M1/M2 macrophage makers in each single cell cluster. (G) Pseudotime trajectory of blood cells and tissue cells. (H) Pseudotime trajectory of nine monocyte-macrophage subpopulations following PAZ@Fe-MOF treatment.

Supplementary Material 8: Table S1. The loading efficiency and encapsulation efficiency of PAZ in PAZ@Fe-MOF.

## Data Availability

No datasets were generated or analysed during the current study.

## Abbreviations

VEGFR: vascular endothelial growth factor receptors
PDGFR: platelet-derived growth factor receptor
c-Kit: mast/stem-cell growth factor receptor
PAZ: pazopanib
scRNAseq: single-cell RNA sequencing
MOF: metal organic framework
PET-CT: positron emission tomography-computed tomography
CCL17: C-C motif chemokine ligand 17
IL10: interleukin 10
TGFβ: transforming growth factor beta
ATF6: activating transcription factor 6
TGFBR1: transforming growth factor beta receptor 1
TXNDC5: thioredoxin domain containing 5
S100a4: S100 calcium binding protein A4
Apoe: apolipoprotein E
Ccr2: C-C motif chemokine receptor 2
Plac8: placenta-specific 8
Ctss: cathepsin S
Crip1: cysteine rich protein 1
ER: endoplasmic reticulum
