# Supplementary figures and images for: Determining M2 macrophages content for the anti-tumor effects of metal-organic framework-encapsulated pazopanib nanoparticles in breast cancer
Source: J Nanobiotechnology. 2024 Jul 20;22:429. doi: 10.1186/s12951-024-02694-z (PMC11264935; doi:10.1186/s12951-024-02694-z)

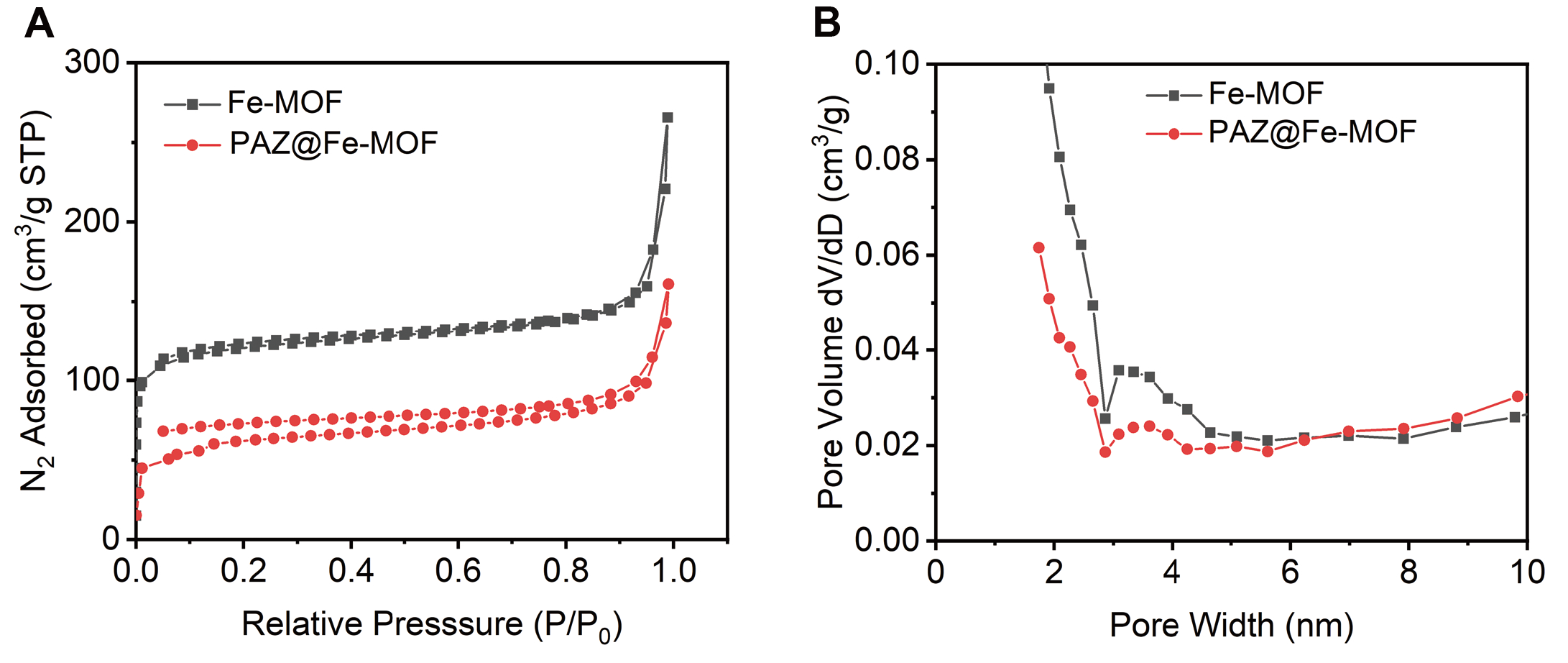

Supplement: Supplementary file 1 — Supplementary Material 1: Figure S1. The porous property of nanoparticles was characterized by N2 absorption and desorption assay. (A-B) The BET surface area (A) and BJH pore size distribution (B) were detected using N2 absorption and desorption assay. [file 12951_2024_2694_MOESM1_ESM.png]

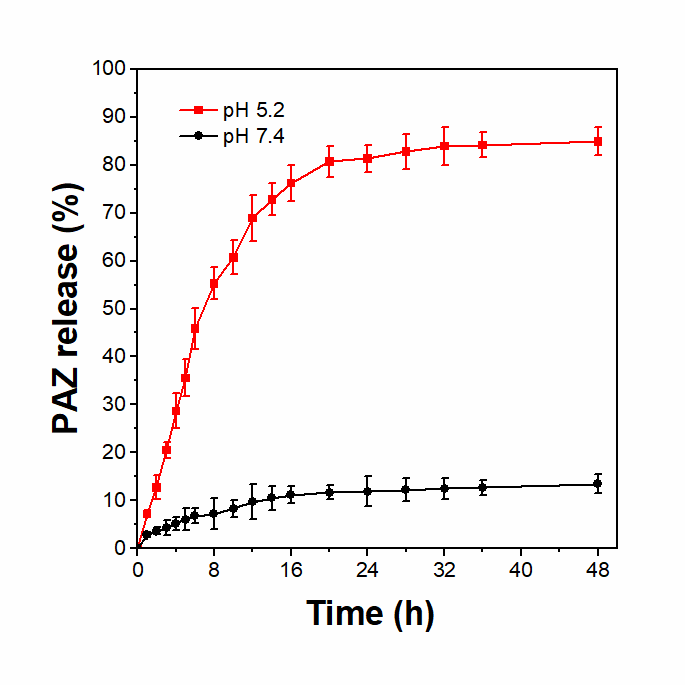

Supplement: Supplementary file 2 — Supplementary Material 2: Figure S2. The release of PAZ from PAZ@Fe-MOF nanoparticles under different pH conditions. [file 12951_2024_2694_MOESM2_ESM.png]

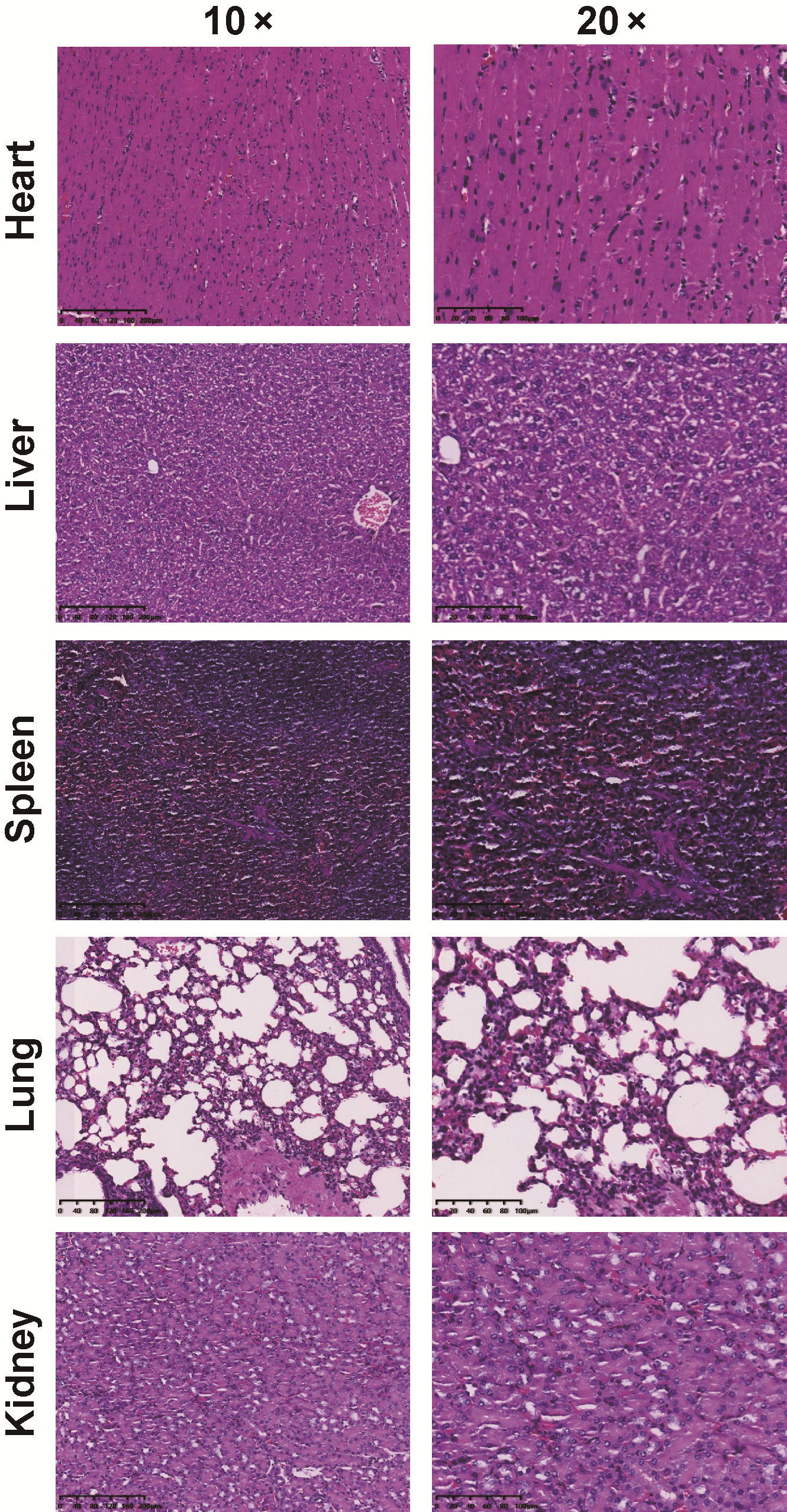

Supplement: Supplementary file 3 — Supplementary Material 3: Figure S3. HE staining indicated the morphological changes of vital organs in orthotopic breast tumor models. [file 12951_2024_2694_MOESM3_ESM.png]

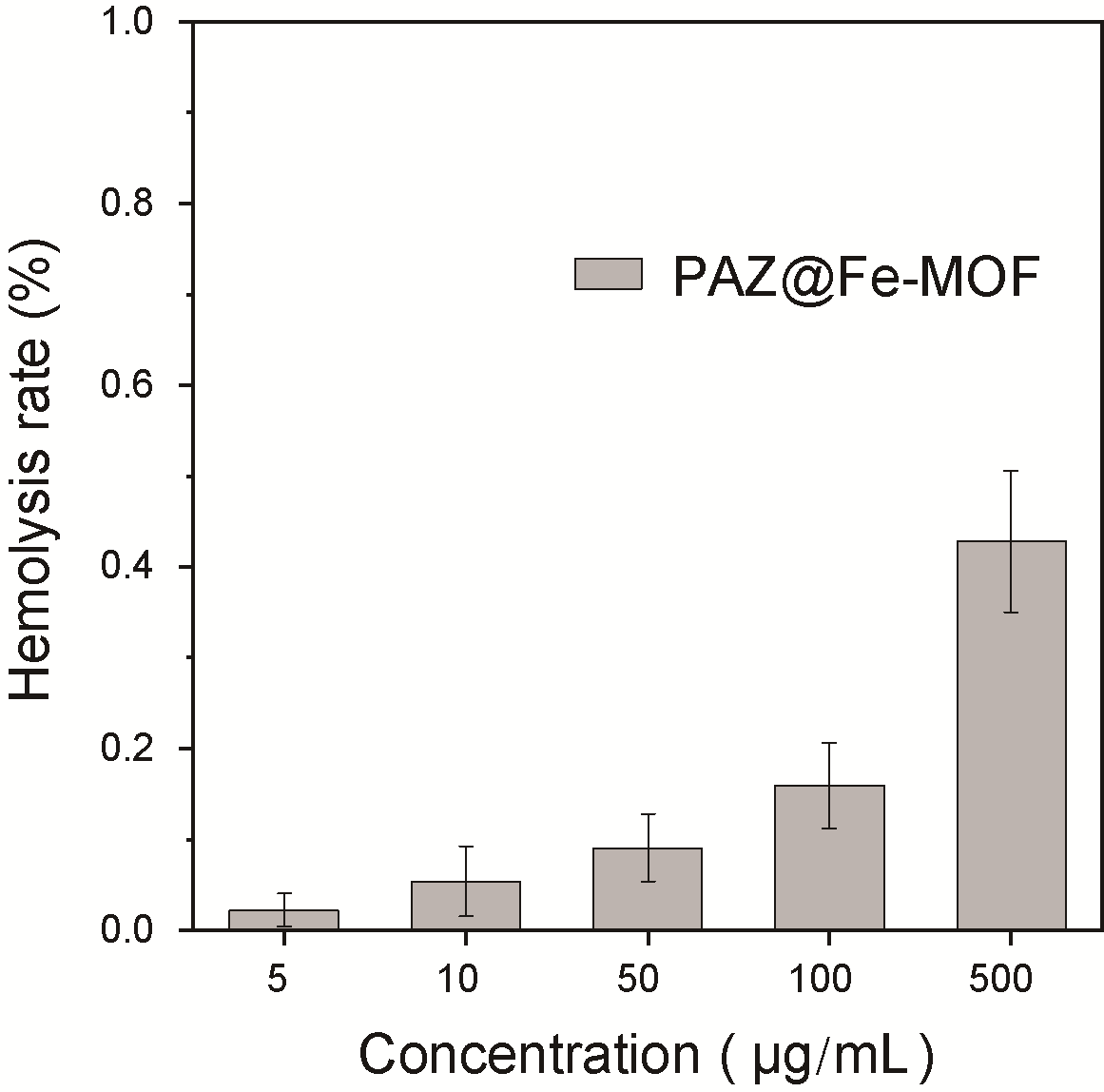

Supplement: Supplementary file 4 — Supplementary Material 4: Figure S4. The hemolysis rate of the PAZ@Fe-MOF to the red blood cells was detected, which reflected the hemolytic compatibility of the prepared nanoparticles. [file 12951_2024_2694_MOESM4_ESM.png]

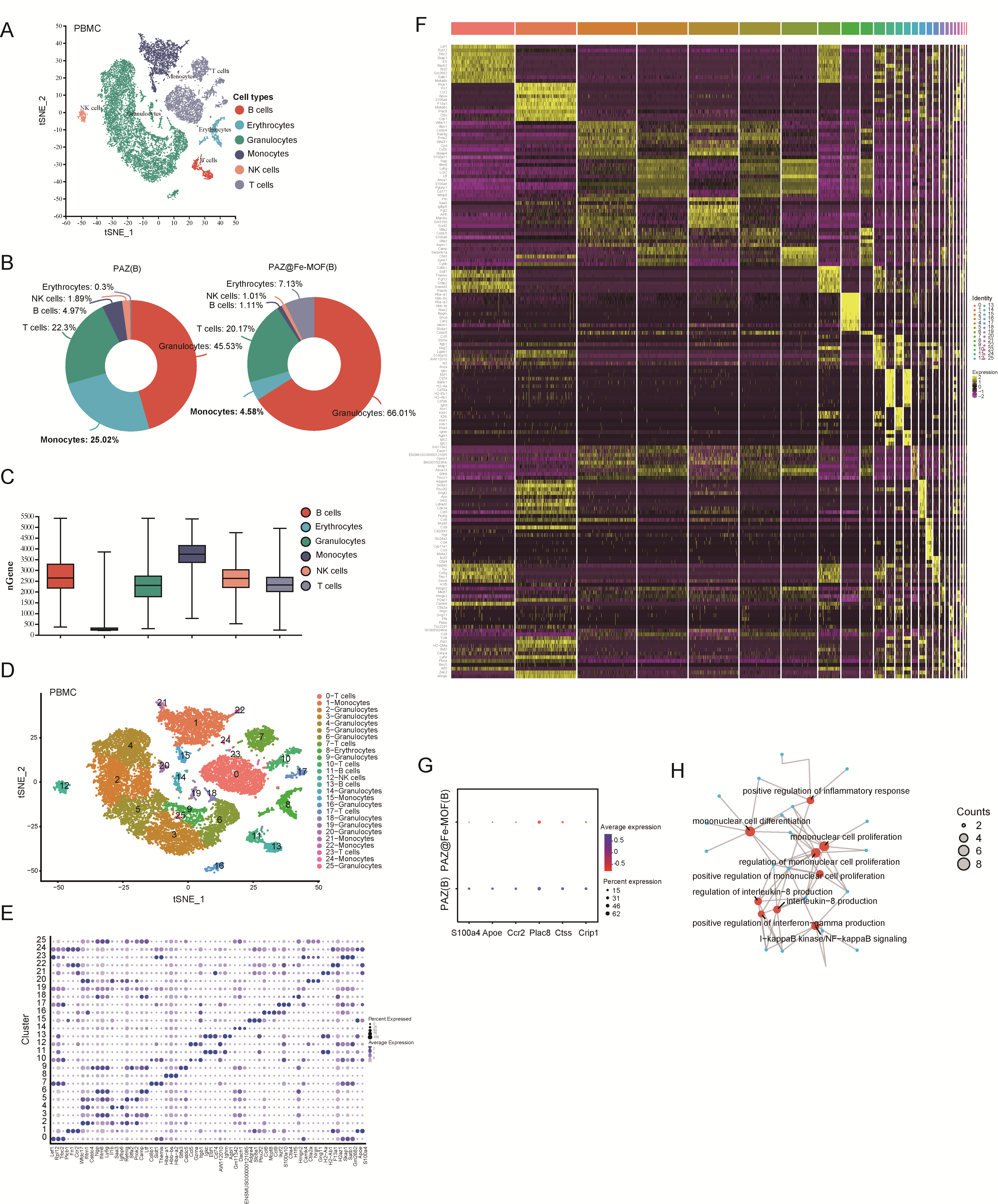

Supplement: Supplementary file 5 — Supplementary Material 5: Figure S5. Single-cell profiling and cell typing in blood. (A) t-SNE visualization of cell types in peripheral blood from the mice treated with PAZ@Fe-MOF and free PAZ. (B) Pie chart indicated the changes of different cell clusters after PAZ@Fe-MOF treatment. (C) The numbers of characteristic genes in different cell clusters. (D) t-SNE indicated the twenty-six clusters from the peripheral blood. (E) Bubble plots indicated the top 3 specific genes in each cluster from the peripheral blood. (F) A heatmap indicated the top 10 specific genes in each cluster from the peripheral blood. (G) Bubble plots indicated the changes of monocytic makers after PAZ@Fe-MOF treatment. (H) The KEGG enrichment analysis of the DEGs between cluster 1 and other clusters. [file 12951_2024_2694_MOESM5_ESM.png]

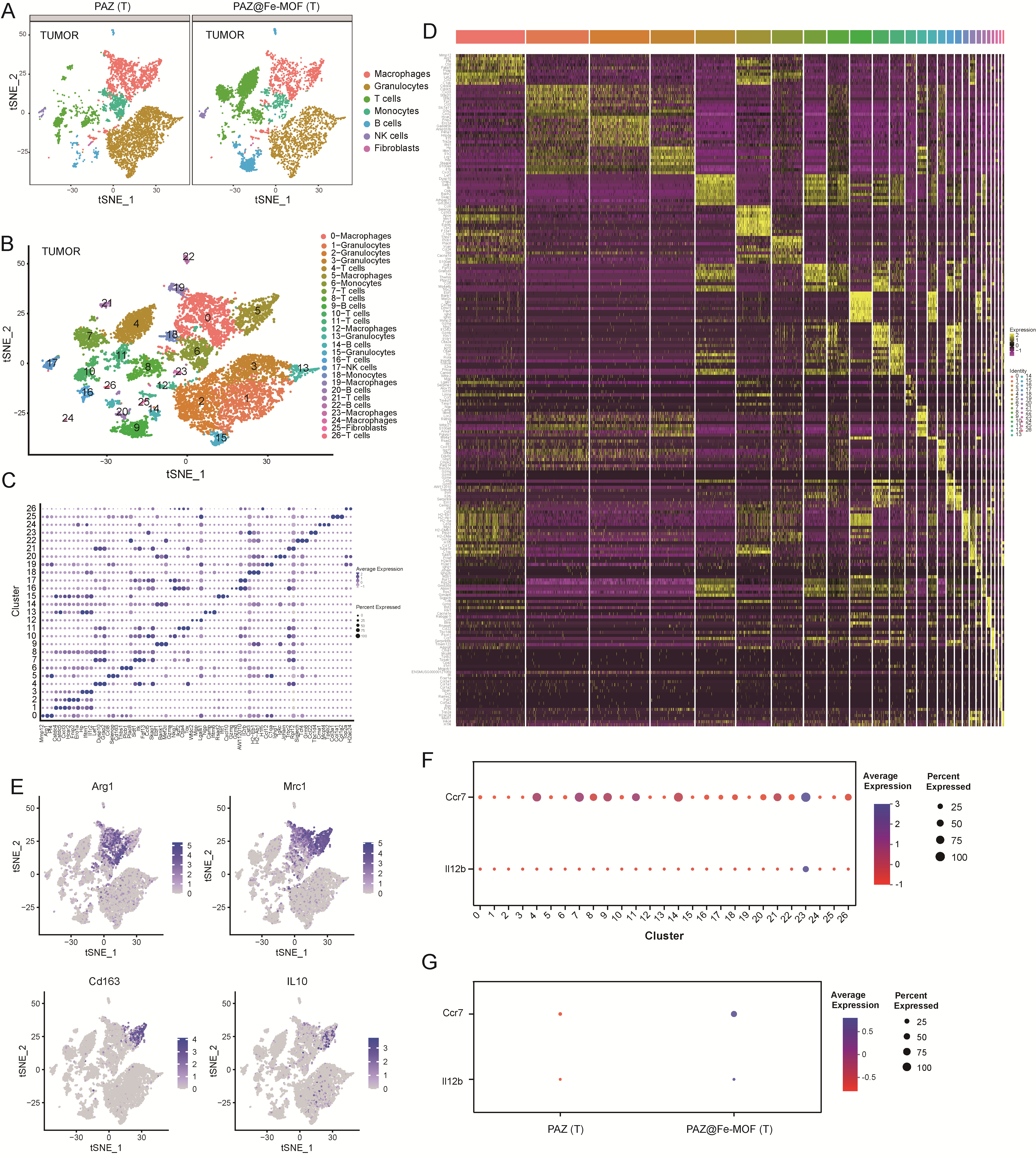

Supplement: Supplementary file 6 — Supplementary Material 6: Figure S6. scRNAseq identified the effect of PAZ@Fe-MOF on M2 macrophages in tissues. (A) t-SNE plots indicated seven cell types from all CD45+ cells. (B) t-SNE indicated the twenty-seven clusters from the tissues. (C) Bobble plot indicated the top 3 specific genes in each cluster from the tissues. (D) A heatmap indicated the top 10 specific genes in each cluster from the tissues. (E) The expression of M2-polarized macrophage makers in each cluster profiled on the t-SNE plots. (F) Bubble plots indicated the expression of M1-like macrophage makers in each single cell cluster. (G) Bubble plots indicated the changes of M1-like macrophage makers after PAZ@Fe-MOF treatment. [file 12951_2024_2694_MOESM6_ESM.png]

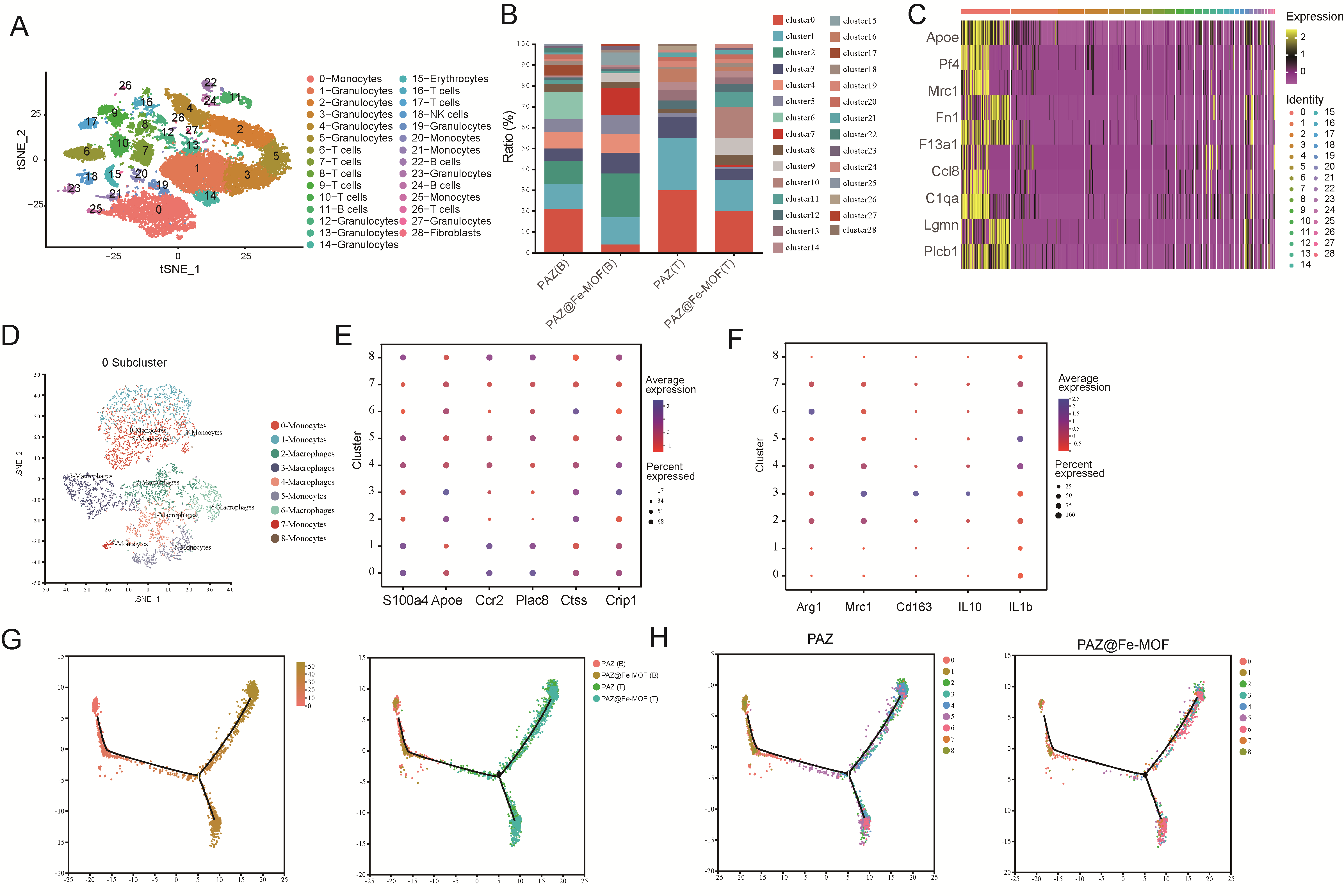

Supplement: Supplementary file 7 — Supplementary Material 7: Figure S7. Reconstructing the pseudotime trajectory of monocyte-macrophage during PAZ@Fe-MOF treatment. (A) t-SNE plots of twenty-nine cell types from blood and tissue specimens. (B) Cell percentages of each cluster were shown in the histograms. (C) The top 10 transcription factors in clusters 0 compared with other clusters. (D) t-SNE plots of nine monocyte-macrophage subpopulations from clusters 0. (E) Bubble plots indicated the expression of monocytic makers in each single cell cluster. (F) Bubble plots indicated the expression of M1/M2 macrophage makers in each single cell cluster. (G) Pseudotime trajectory of blood cells and tissue cells. (H) Pseudotime trajectory of nine monocyte-macrophage subpopulations following PAZ@Fe-MOF treatment. [file 12951_2024_2694_MOESM7_ESM.png]
